# SARS-CoV-2 vaccination and uveitis: Are they linked?

**DOI:** 10.1016/j.amsu.2022.104472

**Published:** 2022-08-28

**Authors:** Summaiyya Waseem, Syed Hassan Ahmed, Sharmeen Fatima, Taha Gul Shaikh, Jawad Ahmed

**Affiliations:** Dow University of Health Sciences, Karachi, Pakistan

**Keywords:** Anterior uveitis, COVID-19 vaccine, Ocular inflammation, Panuveitis, Uveitis

## Abstract

In 2019, the discovery of a new strain of Coronavirus, later referred to as SARS-CoV2 took the world by storm, leading to a pandemic and shutting down all global activities. Several measures were taken adequately to combat the viral havoc, including developing numerous vaccines. All the vaccines currently available for the general population went through rigorous screenings and trials to ensure maximum safety and were only approved after that. However, once they were rolled out in the markets and administered to the population, some adverse reactions were reported, one of which included uveitis. It is an ocular inflammatory condition of the uveal tract, choroid, or iris. If untreated, it can lead to severe consequences, including blindness. It is further divided into four categories based on its anatomical location. Despite the rare incidence of uveitis following COVID-19 vaccination, it may contribute to vaccine hesitancy; hence addressing and digging into the pathophysiological cause is crucial. This study evaluates all the pathophysiological and demographical links between COVID-19 vaccination and uveitis, suggesting appropriate management plans.

## Highlights

• The phenomenon of vaccine associated uveitis has been previously reported with several vaccines.
• The reporting of uveitis following COVID-19 vaccinations might contribute to vaccine hesitancy.
• Past medical history and certain demographic conditions might dispose some people to developing uveitis.
• Corticosteroids were the initial drug of choice for many cases.
• Large scale studies are needed to reach a decisive conclusion.

## 1 Introduction

SARS-CoV-2 is an RNA virus from Wuhan, China, transmitted mainly through respiratory droplets, e.g., coughing or sneezing [1]. The patients commonly present with complaints of fever, cough, and dyspnea, while the lab reports for the majority reveal decreased albumin, elevated C-reactive protein (CRP), lactate dehydrogenase (LDH), erythrocyte sedimentation rate (ESR), and lymphopenia [2]. However, Polymerase Chain Reaction (PCR) continues to be a gold standard diagnostic technique for its detection [3]. As of August 17, 2022, 560 million confirmed cases have been reported, and 6.44 million fell victim to death [4].

To overcome the devastating impacts of the pandemic, widespread efforts and resources were devoted to developing vaccines against the virus. Regulatory authorities have approved and administered numerous vaccines to combat the pandemic. The currently available vaccines stimulate antibody formation against COVID-19 spike protein hence conferring immunity against the virus. The viral vector vaccines, comprising AstraZeneca, Sputnik, and Janssen, employ recombinant DNA technology to incorporate spike protein DNA into the adenovirus. Another category, referred to as mRNA vaccines, injects the messenger RNA for spike protein into the host and includes Pfizer and Moderna [5]. Lastly, vaccines like Sinopharm and Sinovac use a weakened or attenuated virus to generate protective antibodies in the hosts [6].

All the currently available vaccines were approved following critical experimentation and trials and after demonstrating adequate safety profiles [7]. As of December 23, 8.6 billion vaccine doses have been administered [4]. The commonly reported post-vaccination adverse events include pain at the injection site, pyrexia, headache, myalgias, fatigue, and chills, with most of them being transient and self-limited [8]. However, other atypical adverse events have also been reported involving vaccine-induced thrombotic thrombocytopenia (VITT) [9], Guillian Barre Syndrome [10], myocarditis [11], tinnitus [12], and anaphylaxis [13], leading to a massive hesitant response to the vaccine from the public.

More recently, cases of uveitis have been reported following SARS-CoV-2 vaccination, raising a potential association between the two. This study aims to investigate the likely pathophysiology behind COVID-19 vaccine-induced uveitis considering the currently available literature and to highlight evidence-supported clinical approach and management.

## 2 Materials and methods

Two authors (SHA, SW) independently conducted an extensive literature search over PubMed, Google Scholar, and Clinicaltrails.gov from inception till October 6, 2021, without any language restriction. The following keywords, separated by BOOLEAN operators AND and OR, were engaged: “SARS-CoV-2 Vaccine”; “Coronavirus Vaccine”; “Corona Vaccine”; “COVID-19 Vaccine”; “Uveitis”; “Anterior Uveitis”; “Posterior Uveitis”; “Intermediate Uveitis”; “Panuveitis”. Furthermore, gray literature and bibliographies of relevant articles were screened to achieve comprehensive results. Any discrepancies were resolved by discussion with a third reviewer (JA).

Following the studies' selection, two independent authors (TGS, SF) retrieved all the relevant data comprising the author's name, country, patient's age, and sex, past medical history, vaccine administered, time from dose administration till the onset of symptoms, presenting complaint, clinical findings and investigations, treatment, and outcome into a table (Table 1).

**Table 1:** A tabulation of the outcomes of literature review.

Author, year Country	Age Sex	Past Medical History	Vaccine Administered	Time from Vaccination to Onset of symptoms	Presenting Complaint	Clinical Findings and Investigations	Treatment	Outcome
Elsheikh et al. [19], 2021 Egypt	18 y/o Female	Antinuclear antibody (ANA)-positive oligoarticular JIA diagnosed at the age of 7 years and treated with methotrexate till 11-year-old	Sinopharm (2nd Dose)	5 days	Acute bilateral blurred vision, photophobia	BCVA = 6/12 OD and 6/36 OS IOP = 13 OD AND 14 OS Slit lamp = AC inflammation with +2 flare OU and +1 cells OU Anterior segment OCT= Hyperreflective dots in the AC and fine endothelial granularities HLA-B27 = Negative	Topical prednisolone acetate 1% every 2 h and cyclopentolate hydrochloride three times daily	Recovered by week 6
Pan et al. [20], 2021 China	50 y/o Female	Not significant	Inactivated virus vaccine	5 days	Bilateral blurred vision and visual distortion	BCVA = 20/33 OD and 20/66 OS. IOP = 12 mmHg OD and 14 mmHg OS Fundus examination = Optic disc was pale and blurry in right eye and foveal reflex was lost bilaterally After 1-week, visual acuity reduced to 12/200 HLA-B27 = Negative	Triamcinolone acetonide 40 mg via periocular injection and oral prednisone 20 mg once a day	Recovered after 5 weeks
Renisi et al. [21], 2021 Italy	23 y/o Male	Recurrent panic attacks treated with benzodiazepines and developed unilateral periocular erythema with involvement of left eyelid, 5 h after administration of first dose of BNT162b2, treated with topical glucocorticoids for 10 days	BNT162b2 (2nd Dose)	14 days	Unilateral red left eye, conjunctival hyperemia, visual acuity reduction (0.3 logMAR), pain, and photophobia	Slit lamp = pericheratic and conjunctival hyperemia, posterior synechiae, and anterior chamber cells and keratic precipitates in the inferior quadrants	Dexamethasone eye drops three times a day, as well as a cycloplegic agent (atropine 1%) twice daily. After one week of treatment, the glucocorticoid drops were increased to six times a day. After three weeks of symptoms onset, the patient started to improve.	Recovered after 6 weeks
Jain et al. [22], 2021 India	27 y/o Male	Juvenile Idiopathic Arthritis diagnosed at the age of 13 years, single episode of bilateral uveitis in 2012, which subsided with oral and topical steroids	Covishield (1st Dose)	2 days	Unilateral pain and redness in his left eye	VA = 6/6 Slit lamp = Severe circumcorneal OS congestion, 2+ cells in the AC with fine, fresh, non-granulomatous keratic precipitates HLA-B27 = Positive	Topical steroids and cycloplegics	Recovered
Ishay et al. [23], 2021 Israel	28 y/o Male	Behçet's disease, diagnosed four years ago and being maintained on colchicine therapy, 0.5 mg twice daily.	BNT162b2 (1st Dose)	10 days	Left eye pain, redness, and blurred vision	Blood tests showed leukocytosis, elevated CRP and ESR. Ophthalmologic evaluation revealed severe left eye panuveitis compatible with Behçet uveitis.	Pulse IV methylprednisone 1 g/day for 5 days and intensive topical steroid therapy, followed by oral corticosteroids and azathioprine	Recovered
Goyal et al. [24], 2021 India	34 y/o Male	Not significant	Covishield (2nd dose)	9 days	Nasal redness in left eye, progressive floater in right eye progressing to severe vision loss	BCVA: 6/36, N60 (RE), 6/6, N6 (LE) Fundus examination: bilateral multiple yellowish lesions on choroid, clustered at the macula and optic nerve, serous detachments of the retina at multiple locations bilaterally. SD-OCT; RE: Massive subretinal fluid at the macula and smaller serous detachment inferonasal to the optic disc; LE: Small area of subretinal fluid temporal to the optic disc. B-scan ultrasonography: Choroidal thickening in both eyes with RE being 4 times and LE being 2 times than normal. Total leucocyte count = 11200/mm3 (neutrophilic leukocytosis)	Oral prednisolone 100 mg daily, tapering by 10 mg every week	Recovered after 11 days

JIA: Juvenile Idiopathic Arthritis, BCVA: Best Corrected Visual Acuity, OS: Oculus Sinister, OD: Oculus Dextrus, IOP: Intraocular Pressure, OCT: Optical Coherence Tomography, AC: Anterior Chamber, IV: Intravenous, CRP: C-Reactive Protein, ESR: Erythrocyte Sedimentation Rate, SD-COT: Spectral domain-optical coherence tomography, RE: Right Eye, LE: Left Eye.

## 3 Uveitis: what we know about it?

Intuitively, the term “uveitis” refers to the inflammation of the uveal tract comprising the iris, ciliary body, and choroid, but it encompasses a broad spectrum of inflammatory conditions affecting the retina, vitreous humor, and optic nerve as well [14]. Its prevalence varies globally depending on the geographic location and is estimated between 38 and 714 per 100,000 population [15]. Anatomically, uveitis can be classified into four categories: anterior, intermediate, posterior, and pan-uveitis.

Anterior uveitis, the most prevalent type, accounts for approximately 90% of the reported cases in primary care settings and between 50 and 60% in tertiary settings [16]. It is characterized by inflammation of the anterior chamber and may include iritis, iridocyclitis, and anterior cyclitis [16,17]. Intermediate uveitis, the rarest form, is marked by the inflammation of vitreous humor and includes par planitis, posterior cyclitis, and hyalitis. The second most common form, posterior uveitis, is primarily characterized by the inflammation of the retina or choroid. It can further be subclassified into focal, multifocal, and diffuse choroiditis, chorioretinitis, retinochoroiditis, retinitis, and neuro-retinitis. Widespread inflammation involving more than one anatomical zone is classified as pan-uveitis and involves inflammation of the anterior chamber, vitreous humor, choroid, and retina [16,17].

Clinically, it can be divided into three major groups based on the etiologies. Infectious uveitis is more prevalent in developing countries and accounts for 30 to 50% of the reported cases, with the majority manifesting as posterior uveitis or pan-uveitis. Non-infectious causes are more prevalent in developed regions of the world and can be further subdivided depending on their association with or without systemic diseases [16,17]. The last category, masquerade, incorporates other neoplastic and nonneoplastic causes [17].

**Fig. 1:**
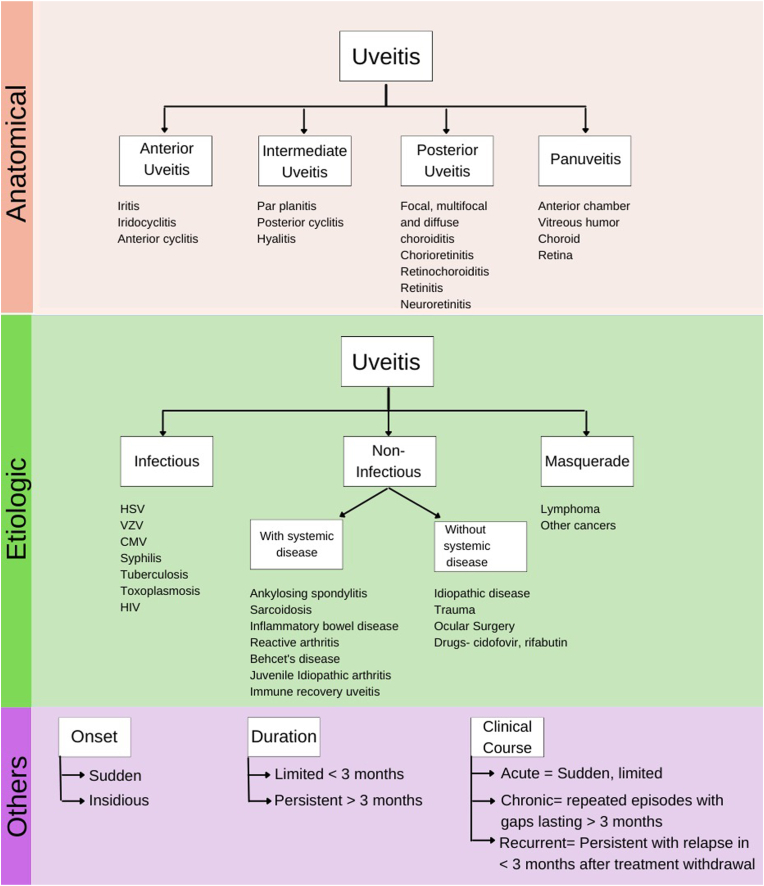
Classifications of uveitis according to international uveitis study group.

Additionally, clinically helpful classifications can be based on the onset, duration, and clinical course. Depending upon the duration, it can be sudden or insidious in onset and either limited or persistent. Uveitis resolving within three months can be labeled ‘*limited*’. It can be referred to as ‘*persistent*’ if it resolves after three months. The disease is defined as acute if it demonstrates a sudden onset and limited duration. In contrast, chronic uveitis is characterized by repeated episodes separated by regular periods without any treatment, lasting more than three months. If persistent uveitis is observed with relapses occurring in less than three months following treatment discontinuation, it can be labeled as recurrent uveitis [16]. Fig. 1 demonstrates the currently available classifications of uveitis and their description.

## 4 Vaccine-associated uveitis

The phenomenon of vaccine-associated uveitis may be rare but is not novel. Numerous cases have been reported following a variety of vaccines, including Bacille Calmette Guerin (BCG), Hepatitis A, Hepatitis B, Influenza, Human papillomavirus (HPV), Varicella, Measles-Mumps-Rubella (MMR), etc. with Hepatitis B vaccine being the major offender and contributing a total of 40% cases. Among the reported case, a female preponderance has been observed, with the mean age of both genders being thirty years. Similar adverse effects have been noticed following simultaneous administration of certain vaccines like Hepatitis A and Hepatitis B combined, MMR and varicella, etc. [18]. More recently, a few cases of vaccine-associated uveitis have been reported following SARS-CoV-2 vaccine administration [[19], [20], [21], [22], [23], [24]].

## 5 Mechanism behind SARS-CoV-2 vaccine associated uveitis

Despite reports of numerous cases, the precise pathophysiology behind this condition is still uncertain. Several hypotheses have been proposed including molecular mimicry between vaccine peptide fragments and uveal self-peptides, delayed-type hypersensitivity with immune complexes deposition, and an immune reaction to vaccination adjuvants. Still, there is an overwhelming need to discern the involved mechanisms. The significant findings of all the reported cases included in this review have been tabulated, as shown in Table 1.

### 5.1 Molecular mimicry

Molecular mimicry refers to the phenomenon whereby a significant degree of resemblance occurs between the foreign and self-peptides. Based on the resemblance between the micro-organisms and hosts’ proteins, it can be classified into four types: (1) Type 1 occurs at the level of proteins. It is characterized by the complete resemblance of a protein not encoded by the micro-organism; (2) Type 2 is determined by homology at the protein level of a protein encoded by the micro-organism; (3) in Type 3 similar amino acid sequence or epitopes are seen; (4) Type 4 is characterized by structural similarities between the microbe or environmental factor and its host [25]. Such morphologic similarities may induce cross-reactivity reactions between antibodies against foreign particles and self-peptides, leading to autoimmunity and tissue damage. The current literature proposes molecular mimicry between proteins from Mycobacterium Tuberculosis and retinal antigens as a potential cause of uveitis following BCG vaccine [26]. According to Kanduc et al. [27], a massive heptapeptide homogeneity exists between the SARS-CoV-2 spike glycoprotein and numerous human proteins. Most of the currently available COVID-19 vaccines employ the genetic code of spike proteins to induce protective antibodies against the Coronavirus [7]. The current literature reports the incidence of multiple autoimmune diseases, including Guillain Barre syndrome [10], vaccine-induced thrombotic thrombocytopenia [9], etc., following SARS-CoV-2 vaccination. Hence, based on the mechanisms behind vaccines induced uveitis, the incidence of autoimmune conditions following COVID-19 vaccines, and the homogeneity between spike protein and human peptides, a cross-reactivity reaction between anti-spike antibodies and uveal peptides may be hypothesized to play a pivotal role in the development of vaccine-associated uveitis. Hence, future studies must scrutinize the molecular similarities between coronavirus spike protein and uveal proteins and investigate potential interactions between anti-spike antibodies and ocular proteins. Furthermore, serologic investigations may contribute significantly towards understanding the involved mechanisms, as such findings were frequently reported in cases of VITT following SARS-CoV-2 vaccination [9].

### 5.2 Immune complex deposition

Another postulated mechanism involves hypersensitivity reaction with immune complex deposition. Immune complex formation is a substantial component of humoral immunity, which involves antibodies binding to antigens. In healthy individuals, these complexes are removed timely via the actions of mononuclear phagocytes [28]. In certain conditions, like systemic lupus erythematosus (SLE), serum sickness, and reactive arthritis, patients develop type III hypersensitivity reaction which involves immune complex deposition within tissues leading to complement activation, mast cells degranulation, the neutrophil influx, inflammation, and ultimately tissue damage [29]. Such hypersensitivity reactions have also been reported following numerous vaccines. The allergic reaction may be generated against either the microbial component or the other components of the vaccines. Although microbial agents’ involvement is rare, the currently available literature suggests hypersensitivity to tetanus and diphtheria toxoids, pneumococcus, and Bordetella pertussis antigens. Similarly, the more commonly involved non-microbial or non-active components may include egg components, gelatin, milk proteins, or adjuvants [30]. The role of immune complex deposition in numerous ocular conditions is well established, and several studies highlight elevated immune complex levels within serum and aqueous in uveitis patients [31]. Based on this, a type III hypersensitivity reaction may be postulated as the underlying mechanism. However, future studies must investigate the role of genetic and environmental factors. Lastly, the involvement of the blood-aqueous barrier and the ocular vascular permeability must also be assessed in patients with SARS-CoV-2 vaccination-associated uveitis, as the currently available literature reports uveal vascular dysregulations and blood-aqueous barrier damage following immune complex infusions in rabbits [32].

### 5.3 Immune reaction to vaccine adjuvants

Adjuvants are a heterogeneous group of chemical substances that enhance the immune system's response to the antigens in the administered vaccine. Based on the mechanism of action, they can be classified into three categories: (1) Inducing depot formation at the injection site like mineral compounds, oil adjuvants, and liposomes. Biodegradable polymer microspheres greater than 10 pm (pm), etc., (2) Functioning as transport vehicles that assists the antigen in targeting immune competent cells and includes biodegradable polymer microspheres lesser than 10 p.m., non-ionic block polymer surfactants, (3) Immune-stimulators including lipopolysaccharide (LPS), Freund's complete adjuvant (FCA), muramyl dipeptide (MDP), lipid A, mono-phosphoryl lipid A (MPL), pertussis toxin (PT), cytokines, etc. [33]. Numerous vaccines, including Hepatitis B, DTaP, MR booster, Hepatitis B booster, flu vaccine, employ a variety of adjuvants. While adding adjuvants ensures an enhanced response and reduces the vaccine production cost, the currently available literature suggests a potential association between adjuvant-containing vaccines and autoimmune and inflammatory adverse events in genetically susceptible individuals [34]. Furthermore, the administration of adjuvants can produce Autoimmune Inflammatory Syndrome induced by Adjuvants (ASIA), also known as Shoenfeld's syndrome. It incorporates several autoimmune and systemic conditions, including sarcoidosis, Sjogren's syndrome, and immune-related adverse effects [35,36].

The current literature suggests adjuvants associated immune reaction as a potential mechanism behind Hepatitis A and yellow fever post-vaccination uveitis, which may result from aluminum-containing adjuvants in hepatitis A vaccine [37,38]. The mRNA and adenovirus vaccines are self-adjuvanted and employ immunogenic liposomes as an adjuvant which serves as a delivery vehicle to achieve high antigen concentration within the target cells [39,40], whereas the protein vaccines or the inactivated ones, like Sinopharm and Sinovac, require adjuvants to achieve an adequate protective response. Both Sinopharm and Sinovac employ aluminum hydroxide [40,41] to enhance their action, which may contribute to vaccine-associated uveitis. Therefore, future studies must investigate the role of adjuvants in SARS-CoV-2 vaccines associated with uveitis and other adverse events. Several HLA haplotypes, including HLA-DBR1, HLA-DQB1, and others, are known to be associated with a variety of ASIA syndromes [36]. Hence, there is an overwhelming need to investigate the genetic and environmental involvement in the reported cases, as this may assist in redefining the criteria for COVID-19 vaccine administration.

### 5.4 Toll-like receptors (TLRs)

Recognition of microbial Pathogen-Associated Molecular Patterns (PAMP) by the host's germline-encoded Pattern Recognition Receptors (PRR) is an integral component of innate immune response initiation [42]. TLRs, a family of pattern recognition receptors, are type I transmembrane proteins that comprise an ectodomain, a transmembrane region, and cytosolic toll-IL-1 receptor (TIR) domains that stimulate the signaling pathways leading to the secretion of inflammatory cytokines, type I IFN, chemokines, and antimicrobial peptides [43]. The ocular tissue predominantly expresses the TLR-4 category of the TLRs, primarily in the uvea, retina, and sclera, which upon stimulation by PAMPs like lipopolysaccharide (LPS) may result in inflammation of a variety of ocular tissues. The study by Li et al. [44], proposes the potential involvement of viral RNA and spike protein as PAMPs in inducing an innate immune response against the SARS coronaviruses. Therefore, future studies must investigate potential interactions between ocular TLRs and PAMPs like molecules found within the coronavirus vaccines, and what factors predispose specific individuals to develop adverse events while the majority do not.

### 5.5 Past medical history

Two of the included studies reported cases with a known history of Juvenile Idiopathic Arthritis (JIA) [19,22]. It is the most prevalent chronic childhood rheumatic disease of unknown etiology, presenting with a diverse spectrum of clinical manifestations most commonly involving peripheral arthritis. Although the mechanism behind the disease is still unknown, it involves an exaggerated immune response to endogenous or exogenous antigens [45]. The disease often presents with extra-articular manifestations like uveitis. The prevalence of JIA-associated uveitis varies from region to region. Numerous risk factors like age, gender, JIA type, antinuclear antibody (ANA), and HLA-B27 positivity are associated with it [46]. Despite many instances of JIA-associated uveitis reported in the literature, its precise pathophysiology is still ambiguous. However, due to JIA's association with HLA, an autoimmune reaction involving intra-ocular antigens like retinal S-antigen, retinol-binding protein 3 (RBP3), and tyrosinase-related proteins, may be suspected [46,47]. Therefore, an exaggerated immune response to particular vaccine components may further exacerbate the underlying disease, leading to uveitis.

Another study [23] included in the review, reported the incidence of vaccine-associated uveitis in a patient diagnosed with Behçet's disease. It is a chronic, relapsing, multisystemic disorder that presents with a variety of clinical manifestations [48]. While it can involve numerous systems, the ocular system is the most frequently affected. It can lead to uveitis, cataract, glaucoma, posterior segment involvement with vasculitis, vitritis, retinitis, pan-uveitis, retinal edema, cystoid macular degeneration, venous or arterial occlusion, disc edema, and retinal detachment [48,49]. Although its etiology is still unknown, it involves an auto-inflammatory reaction triggered by infectious or environmental factors in genetically susceptible patients. Therefore, the possibility of vaccine components triggering such a reaction cannot be eliminated and requires further investigation.

However, more large-scale studies are required to draw a significant association between JIA or Behçet's disease and SARS-CoV-2 vaccine-associated uveitis.

### 5.6 Overview

Despite several cases of SARS-CoV-2-associated uveitis being reported, the precise pathophysiology is still not elucidated. Although numerous potential hypotheses have been proposed, there is an overwhelming need for further studies to discern the underlying mechanisms. Furthermore, the incidence of COVID-19 vaccine-associated uveitis only in particular individuals indicates the involvement of other genetic and environmental predispositions, making them susceptible to such reactions. Future studies must emphasize determining any such predispositions, as it may help redefine the vaccine administration criteria and reduce the incidence of uveitis and other adverse events.

## 6 Demographics

**Fig. 2:**
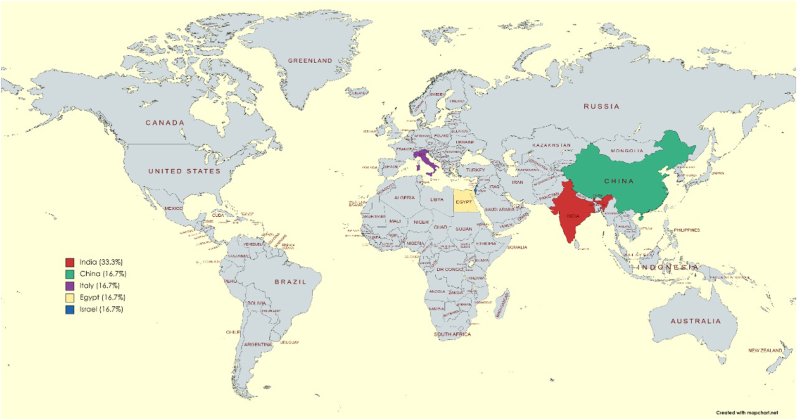
Geographical distribution of the reported cases.

This review scrutinizes data from six patients (four males, two females) with a mean age of 30.0 ± 10.2 years. Fig. 2 demonstrates the geographical distribution of the reported cases around the globe, with a predominance observed in the Asian continent. Out of the six reported cases, two were presented in India, while one case each was reported from China, Italy, Israel, and Egypt. Based on the geographical and gender distribution of the reported cases, future studies must emphasize establishing links between the various genetic and environmental factors predisposing individuals to such adverse events.

## 7 Diagnosis

**Fig. 3:**
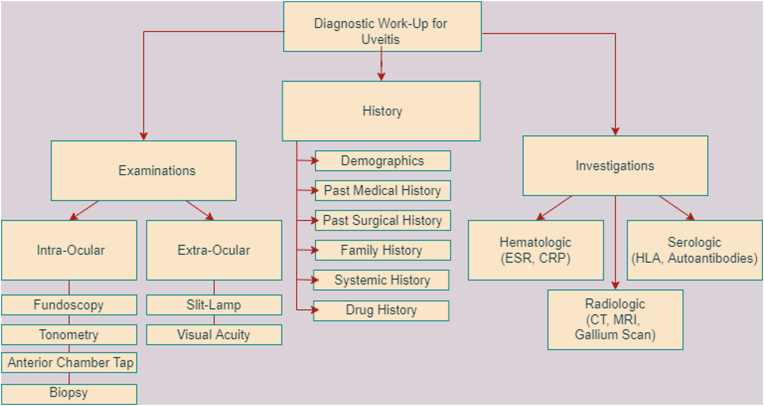
Diagnostic work-up for uveitis.

The diagnostic approach to uveitis varies based on its etiology. However, to reach a submissive conclusion, a systematic approach is crucial. Fig. 3 outlines the key work-up necessary to establish a differential diagnosis of uveitis [[50], [51], [52]].

## 8 Treatment

Dun et al. recommend a stepwise treatment approach, starting from corticosteroids and moving forward to immunosuppressive agents, as needed [53]. Numerous studies have demonstrated the use of other agents as well.

Herein, we have enlisted the treatment options for uveitis with their exact role and importance in the management.

### 8.1 Corticosteroids

Corticosteroids inhibit the enzyme phospholipase A2, ultimately inhibiting the Arachidonic Acid release, thus preventing the release of inflammatory mediators [54]. It is considered the most potent first-line treatment for non-infectious uveitis. Most of the included cases administered topical forms; however, oral and Intravenous (IV) forms are also available. Topical corticosteroids, mainly prednisolone 1% or dexamethasone 0.1%, are more effective in anterior uveitis as they can only penetrate the anterior segment of the eye [55]. In intermediate and posterior uveitis, periocular sub-tenon injections help achieve the desired effect.

Oral prednisone, at an initial dose of 1–2 mg/kg is usually the drug of choice in severe ocular inflammation. In widespread ocular inflammation, IV methylprednisolone at 30 mg/kg shall be administered with oral corticosteroid therapy [54,56]. Despite that corticosteroids provide immediate ocular inflammation relief, there are numerous adverse effects of their long-term use, including Cushingoid changes, growth retardation in children, peptic ulceration, hyperglycemia, cataract, glaucoma, and visual impairment [54,57]. Hence, it is crucial to taper off and discontinue their use with time to avoid such consequences.

### 8.2 Methotrexate

Beneficial in chronic uveitis [58], Methotrexate inhibits the conversion of dihydrofolate to tetrahydrofolate [59]. Belonging to the class of anti-metabolites, this drug has a slow onset of action, hence requiring six months to reach the same effects as corticosteroids [60]. Reportedly, it was first used for ocular disorders in 1965 [59]. The initial recommended dose is 7.5 gm once a week on oral administration and then can be adjusted up to 15 mg/week gradually [57].

Methotrexate is more effective in the pediatric age group compared to adults [56]. Though usually well tolerated, it has its set of adverse events, including hepatotoxicity, bone marrow suppression, and interstitial pneumonia [57].

### 8.3 Azathioprine

Belonging to the class of anti-metabolite, Azathioprine exerts its anti-inflammatory effects by inhibiting the purine synthesis [61]. It converts into its active metabolites, mercaptopurine (6-MP) and thioguanine (6-TGN), which incorporates into DNA, hence inhibiting the division [61]. It is prescribed orally at a dose of 1–2 mg/kg daily with therapeutic results seen after 1–3 months [54] and is usually a drug of choice in the adult population [62]. Severe reactions including hepatotoxicity, myelosuppression with leukopenia and thrombocytopenia, and malignancies have been reported after its use [54,62].

### 8.4 Mycophenolate Mofetil

Administered at an oral dose of 0.5–1 g [56,57], Mycophenolate Mofetil is a prodrug of mycophenolic acid and interferes with the de novo purine synthesis pathway which is essential in the proliferation of B and T lymphocytes [57,62,63]. It is given orally with regular monitoring of CBC and LFT [57,62]. This drug is effective in both children and adults either alone or in combination with other steroid-sparing agents [60]. Adverse events like leukopenia, lymphocytopenia, elevated liver enzymes, and sepsis are less common [57,62].

### 8.5 Infliximab

First approved in 1998 by FDA, Infliximab is a chimeric Tumor Necrotic Factor Alpha (TNF-α) monoclonal antibody [64]. It is administered as an IV at a dose of 5–10 mg/kg [62]. In uveitis secondary to Bechet's disease, it has shown tremendous results [65]. In a trial by Suhler et al., 23 patients were given infliximab with other immunosuppressive drugs, and the results were affirmative [66]. However, this drug has been discontinued owing to a significant number of reported adverse effects.

### 8.6 Adalimumab

Given as a subcutaneous injection at 40 mg dose [65], Adalimumab is a fully-humanized recombinant anti-TNF-α monoclonal antibody. In recent years, adalimumab has significantly reduced ocular inflammation in uveitis entities like Bechet's disease and HLA-B-27-related uveitis [67,68].

In a trial by Sheppard et al., all the patients with uveitis showed clinically significant results when administered this drug [69].

Numerous other drugs are also prescribed for uveitis, which are summarized in Table 2. However, it is worth noting that Food and Drug Administration (FDA) has only approved corticosteroid eye drops for anterior and sustained-release corticosteroid implants for intermediate, posterior, and panuveitis [53]. All other treatment agents are used off label [53]. Moreover, all cases only preferred corticosteroids. While one plausible explanation lies in the difference in etiologies, more data is still necessary to cross-check their efficacy with other available options.

**Table 2:** Other treatment options for uveitis.

Class	Drug	Use/Side Effects
TNF Alpha Inhibitor	Etanercept (Discontinued)	Initial studies showed that etanercept may be used to treat uveitis, but later controversies began, and now, It's not recommended for uveitis [62,65].
	Golimumab	Multiple case reports and case series have proved its effectiveness in uveitis especially where other anti-TNF- α agents failed to achieve the desired goals [70].
	Certolizumab	Studies have shown that certolizumab can be an alternate anti-TNF-α therapy in chronic uveitis [71].
Fusion Protein (Immuno-modulator)	Abatacept	In some cases of JIA-associated uveitis, this drug has shown effective results in reducing ocular inflammation, where other immunosuppressive agents were failed to achieve the desired effect [56].
CD-20 Inhibitor	Rituximab	This drug has been used to control ocular inflammation where other treatment options are ineffective, with no significant side effects or any risk of infection [72].
IL Receptor Antagonist	Anakinra (IL-1)	This drug was found to be a safe option for treating Bechet's disease-related uveitis [73].
	Tocilizumab (IL-6)	Several studies have demonstrated the effectiveness of this drug in recalcitrant uveitis [74,75].
	Daclizumab (IL-2)	Daclizumab may have shown beneficial effects in some cases of uveitis, but its use is considered off-label [62].

TNF: Tumor Necrosis Factor; CD: Cluster of Differentiate; IL: Interleukin.

## 9 Conclusion

This study outlines the precise plausible pathophysiology of vaccine-associated uveitis and its potential management strategy. Although the benefits of COVID-19 vaccination precede the potential health threats, there is a need to evaluate and thoroughly investigate any reported adverse event's co-occurrence with the vaccine administration, like uveitis. Moreover, pre-eminent emphasis should be placed on crafting adequate, universally-comprehensive, treatment approaches alongside rigorous efforts to find missing pathophysiological and demographic in prospective trials. This will aid in reducing vaccine hesitancy and fostering more promising results for the global vaccination program.

## Ethical approval

No ethical approval required since it is a review article consisting of publicly available data.

## Sources of funding

No funding sources.

## Author contributions

Syed Hassan Ahmed: Study concept and design along with writing.

Summaiyya Waseem: Study concept and design along with writing.

Sharmeen Fatima: Writing the manuscript.

Taha Gul Shaikh: Writing the manuscript and reviewing.

Jawad Ahmed: Critical reviewing and suggestions.

## Registration of research studies

Name of the registry: None.

Unique Identifying number or registration ID: None.

Hyperlink to your specific registration (must be publicly accessible and will be checked): None.

## Guarantor

All authors take full responsibility of the article.

## Consent

No Consent is required for review articles.

## Ethics statement

Not applicable.

## Funding

None.

## Provenance and peer review

Not commissioned, externally peer-reviewed.

## Declaration of competing interest

No conflict of interest to declare by any author.
